# Beliefs about pregnancy and its implications regarding self-care in the diet of a population group from Montería, Córdoba

**DOI:** 10.1192/j.eurpsy.2022.2230

**Published:** 2022-09-01

**Authors:** E.P. Ruiz Gonzalez, J. Velez Carvajal, A. Uribe Urzola, M. Muñoz Argel, M. Quintana Fernandez

**Affiliations:** Universidad Pontificia Bolivariana, Cordoba, Monteria, Colombia

**Keywords:** food, beliefs, self-care, Pregnancy

## Abstract

**Introduction:**

Pregnancy has sociocultural implications that lead to conceiving it depending on the cultural context (Noguera & Rodríguez, 2008). Self-care is a cultural practice associated with the well-being of mother and child during pregnancy (Carmona, Hurtado and Marín 2007). Being relative to culture, self-care varies according to current beliefs. Beliefs are the concretion of a way of thinking about the environment that surrounds us (Peirce, 1903).

**Objectives:**

Analyze the beliefs of women from a population group in Montería, about pregnancy and its implications regarding self-care in eating.

**Methods:**

Qualitative approach. Sources: primary. Sample: due to saturation, 15 pregnant women assigned to the Mocarí neighborhood hospital in Montería. Instrument: semi-structured open interview. The information was processed through AtlasTi, implementing content analysis. Emerging categories: contents, routines in food.

**Results:**

Main belief: food affects the well-being of mother and child. It is adequate or inappropriate depending on categories such as content and routines. The former refer to the food consumed, the latter indicate the times of consumption.

**Figure:**
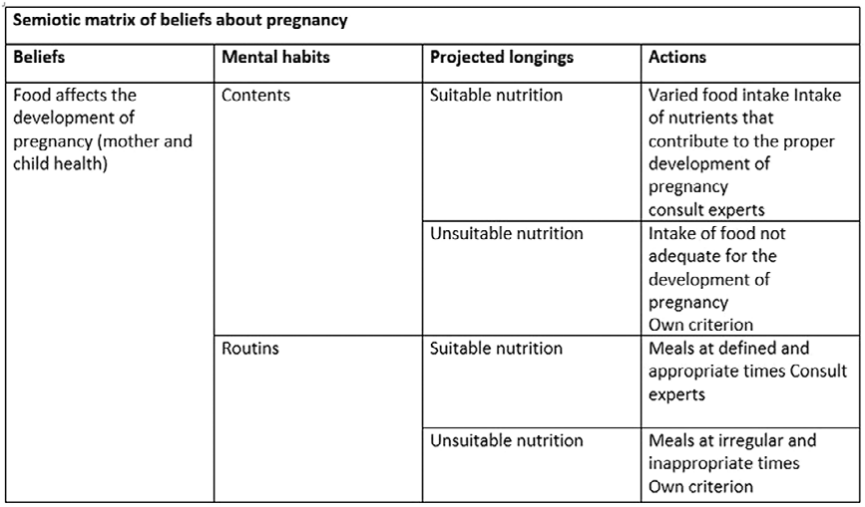

**Conclusions:**

Beliefs about pregnancy operate as generators of mental habits, projected wishes and concrete actions. Therefore, they are an important starting point for the implementation of self-care practices at the institutional level.

**Disclosure:**

No significant relationships.

